# Anti-erythrocyte antibodies may contribute to anaemia in *Plasmodium vivax* malaria by decreasing red blood cell deformability and increasing erythrophagocytosis

**DOI:** 10.1186/s12936-016-1449-5

**Published:** 2016-08-04

**Authors:** Luiza Carvalho Mourão, Paula Magda da Silva Roma, Jamila da Silva Sultane Aboobacar, Camila Maia Pantuzzo Medeiros, Zélia Barbosa de Almeida, Cor Jesus Fernandes Fontes, Ubirajara Agero, Oscar Nassif de Mesquita, Marcelo Porto Bemquerer, Érika Martins Braga

**Affiliations:** 1 Departamento de Parasitologia, Universidade Federal de Minas Gerais, Belo Horizonte, MG Brazil; 2 Departamento de Física, Universidade Federal de Minas Gerais, Belo Horizonte, MG Brazil; 3 Faculdade de Ciências Médicas, Universidade Federal do Mato Grosso, Cuiabá, MT Brazil; 4 Embrapa Recursos Genéticos e Biotecnologia, Brasília, DF Brazil

**Keywords:** *Plasmodium vivax*, Anaemia, Non-infected RBC, Auto-antibodies, Erythrophagocytosis, Defocusing microscopy

## Abstract

**Background:**

*Plasmodium vivax* accounts for the majority of human malaria infections outside Africa and is being increasingly associated in fatal outcomes with anaemia as one of the major complications. One of the causes of malarial anaemia is the augmented removal of circulating non-infected red blood cells (nRBCs), an issue not yet fully understood. High levels of auto-antibodies against RBCs have been associated with severe anaemia and reduced survival of nRBCs in patients with falciparum malaria. Since there are no substantial data about the role of those antibodies in vivax malaria, this study was designed to determine whether or not auto-antibodies against erythrocytes are involved in nRBC clearance. Moreover, the possible immune mechanisms elicited by them that may be associated to induce anaemia in *P. vivax* infection was investigated.

**Methods:**

Concentrations of total IgG were determined by sandwich ELISA in sera from clinically well-defined groups of *P. vivax*-infected patients with or without anaemia and in healthy controls never exposed to malaria, whereas the levels of specific IgG to nRBCs were determined by cell-ELISA. Erythrophagocytosis assay was used to investigate the ability of IgGs purified from each studied pooled sera in enhancing nRBC in vitro clearance by THP-1 macrophages. Defocusing microscopy was employed to measure the biomechanical modifications of individual nRBCs opsonized by IgGs purified from each group.

**Results:**

Anaemic patients had higher levels of total and specific anti-RBC antibodies in comparison to the non-anaemic ones. Opsonization with purified IgG from anaemic patients significantly enhanced RBCs in vitro phagocytosis by THP-1 macrophages. Auto-antibodies purified from anaemic patients decreased the nRBC dynamic membrane fluctuations suggesting a possible participation of such antibodies in the perturbation of erythrocyte flexibility and morphology integrity maintenance.

**Conclusions:**

These findings revealed that vivax-infected patients with anaemia have increased levels of IgG auto-antibodies against nRBCs and that their deposition on the surface of non-infected erythrocytes decreases their deformability, which, in turn, may enhance nRBC clearance by phagocytes, contributing to the anaemic outcome. These data provide insights into the immune mechanisms associated with vivax malaria anaemia and may be important to the development of new therapy and vaccine strategies.

## Background

Anaemia is the most common clinical manifestation and a major complication in patients with vivax malaria [1–5]. Although the development of the parasite inside the erythrocytes directly promotes the destruction of red blood cells (RBCs), this mechanism by itself does not explain anaemia, especially for *Plasmodium vivax*, which infects reticulocytes [6]. Indeed, the parasite burden does not always correlate with the severity of the infection in endemic areas [7]. Furthermore, the destruction of RBCs persists during the convalescence phase [8] or even for weeks after treatment [9–11]. These observations suggest that the destruction of non-infected red blood cells (nRBCs), an issue not yet fully understood, may play an important role in vivax anaemia.

Auto-antibodies probably play a role in the destruction of nRBCs. The presence of these circulating immunoglobulins has been well documented in both *Plasmodium falciparum* and *P. vivax* infections, being occasionally associated with the degree of severity of the disease [12–16]. Higher levels of auto-antibodies against RBC proteins have been shown in *P. vivax*-infected patients in comparison with *P. falciparum* infection [13]. However, whether or not auto-antibodies are involved in anaemia and the possible immune mechanisms elicited by them to induce anaemia in vivax malaria remains unknown.

This study investigated the hypothesis that the recognition of RBC surface proteins by IgG auto-antibodies induced during vivax malaria leads to the opsonization of nRBCs, facilitating their removal by erythrophagocytosis. The role of these auto-antibodies in the destruction of nRBCs was determined by investigating their ability to enhance in vitro phagocytosis promoted by macrophages that were differentiated starting from THP-1 cells. Finally, defocusing microscopy (DM), a non-invasive and powerful optical microscopy technique, was used to assess the effects of these auto-antibodies in the biomechanical properties of the nRBC membrane. This is the first report to show that IgG auto-antibodies produced during vivax malaria change the nRBC membrane fluctuation dynamics, increasing the rigidity of these cells. The increased deposition of self-antibodies on the surface membrane of nRBCs may accelerate the clearance of non-infected erythrocytes leading to anaemia during *P. vivax* infection.

## Methods

### Patients

*Plasmodium vivax*-infected patients between 18 and 68 years old were selected among individuals presenting with classic symptoms of malaria, who sought medical care in the Hospital Universitário Júlio Muller, in Cuiabá (Mato Grosso State), Brazil, between February 2006 and January 2008. They were examined and interviewed by a trained physician, who applied a questionnaire to obtain demographic and epidemiological information. It is important to mention that active malaria transmission does not occur in Cuiabá. Since patients reported short visits to other areas in the Brazilian Amazon where malaria is endemic, it is possible that they had become infected there. Blood was collected by vein puncture into EDTA tubes and used for complete blood count and also to obtain plasma samples, which were separated by centrifugation and stored at −20 °C until experiments were performed. *P. vivax* mono-infections were diagnosed by thick blood smear and further confirmed by nested PCR amplification of species-specific sequence of the 18S SSU rRNA gene of *Plasmodium* as previously described [17]. All patients with malaria were treated according to the Brazilian Ministry of Health guidelines for malaria therapy. Based on the laboratory results of complete blood count, patients were assigned into two groups: (i) malaria patients without anaemia (n = 119); and (ii) malaria patients with anaemia (n = 11) (Table 1). For the current study, anaemia was set as haemoglobin levels less than or equal to 11 g/dL and only patients with normocytic (mean corpuscular volume 80–96 fL) and normochromic (mean corpuscular haemoglobin concentration 32–36 g/dL) anaemia were included. Patients showing signs of severe malnutrition or who were infected with HIV or hepatitis virus were excluded from the study. As controls, sera from malaria-naïve volunteers (n = 11) who lived in a non-endemic area (Belo Horizonte, Minas Gerais State, Brazil) and who had never been exposed to malaria were included. Written informed consent was obtained from each volunteer prior to blood collection. Ethical clearance was provided by the Ethics Committee of the National Information System on Research Ethics Involving Human Beings (SISNEP-CAAE01496013.8.0000.5149).

**Table 1:** Description of the study population

Characteristic	*P. vivax* anemic (n = 11)	*P. vivax* non-anemic (n = 119)	p value
Age (years)	32 [19–52]	39 [27–50]	0.6846
Number of malaria previous episodes	2 [1–5]	0 [0–5]	0.2589
Parasitemia (parasites/µL)	1872 [1030–5669]	5061 [1050–8364]	0.3559
Hemoglobin (g/dL)	10.25 [9.15–10.68]	13.34 [12.50–14.50]	<0.0001
Hematocrit (%)	31.45 [30.78–33.33]	40.0 [37.28–44.08]	<0.0001
Leucocytes (cells/mm^3^)	5950 [4525–7475]	5400 [4400–6800]	0.5317
Platelets (cells/mm^3^)	92,500 [52,750–133,750]	106,000 [78,000–150,000]	0.2979

The values are shown as median and interquartile ranges. p values were calculated using Mann–Whitney tests

### Detection of total IgG

Serum levels of total IgG were determined by sandwich ELISA. A 96-well, flat-bottomed, polystyrene microplate (Corning Incorporation, Corning, NY, USA) was coated with purified sheep polyclonal anti-human IgG (Sigma-Aldrich, St Louis, MO, USA) diluted 1:4000 in 0.1 M carbonate buffer, pH 9.6. Each serum sample, diluted 1:100 in phosphate buffered saline (PBS) containing 0.05 % Tween 20, was added in duplicate to the plate and horseradish peroxidase (HRP)-conjugated polyclonal anti-human IgG (Sigma-Aldrich) was used at 1:2000 dilution. Binding was revealed using 0.5 mg/mL *o*-phenylenediamine dihydrochloride (OPD) substrate (Sigma-Aldrich) in 0.05 M phosphate–citrate buffer, pH 5.0 and the reaction was stopped with 3 M H_2_SO_4_. Optical density (OD_492 nm_) was determined in a Spectra Max 250 microplate reader (Molecular Devices, Sunnyvale, CA, USA). Levels of total IgG for each serum were calculated by interpolation of absorbance readings from a standard calibration curve obtained using 0.001 to 1 mg/mL of purified human IgG (Sigma-Aldrich).

### Cell-ELISA for detection of anti-nRBC IgG

To detect IgG against surface proteins of non-infected RBCs, each well of a 96-well, flat-bottomed, polystyrene microplate (Corning Incorporation, Corning, NY, USA) was coated with approximately one million RBCs (obtained from a healthy non-exposed individual whose blood type is O^+^) diluted in PBS containing 1 % (w/v) BSA (PBS/BSA), following an overnight incubation at 4 °C. After five washes with PBS the plate was blocked with 5 % BSA for 2 h at 37 °C. Plates were washed again and were incubated with sera samples in duplicate diluted 1:100 in PBS/BSA for 2 h at 37 °C. Wells were washed again and then incubated with HRP-conjugated anti-human IgG antibody (Sigma-Aldrich) diluted 1:500 in PBS/BSA for 90 min at 37 °C. The binding was revealed with OPD substrate as described in the section ‘Detection of total IgG’. The levels of specific IgG were expressed as reactivity index (RI), which was calculated as the ratio between the mean optical density (OD) by each sample duplicate and the mean OD plus three standard deviations of samples from 11 malaria-naïve volunteers never exposed to malaria.

### IgG purification

For further experiments, sera pools of each studied group were prepared and IgGs were purified from them by affinity chromatography using prepacked HiTrap™ Protein A HP columns (GE Healthcare, Wauwatosa, WI, USA) according to the manufacturer’s recommendation. Eluted fractions were analysed for protein content by measuring absorption at 280 nm in a spectrophotometer and those containing IgGs were pooled together and dialysed overnight at 4 °C against PBS with at least three buffer changes.

### Erythrophagocytosis assays

Human monocytic leukaemia THP-1 cells were grown (5 % CO_2_ at 37 °C) in RPMI 1640 (Merck–Millipore, Darmstadt, Germany) containing 10 % heat-inactivated fetal bovine serum (FBS, Sigma-Aldrich), 10,000 U/mL penicillin, 10 mg/mL streptomycin, 25 µg/mL amphotericin B, l-glutamine and HEPES, at a density below 1.0 × 10^6^ cells/mL. Differentiation of THP-1 cells was achieved after incubation with 100 nM phorbol-12-myristate-13-acetate for 72 h. Erythrocytes from the same blood donor used for cell-ELISA were opsonized by incubation of 1.0 × 10^7^ erythrocytes/mL with 2 % v/v of purified IgG from the serum pool from each studied group or with commercial antibodies such as polyclonal anti-human RBC IgG. Opsonized RBCs were added to each LabTek chamber (Merck–Millipore) containing 1.0 × 10^6^/mL activated THP-1 monocytes and incubated, at 37 °C, for 2 h. Non-phagocytized erythrocytes were lysed with ultrapure water. The chambers were dried and stained with Giemsa (Merck–Millipore). Phagocytosed RBCs were counted on 400 phagocytic cells and expressed as ingestion index (I index).

I = (ingested RBC/phagocytic cells) × 100.

### Defocusing microscopy (DM)

The impact of antibodies in the amplitude of membrane fluctuations of single nRBC were examined by DM. In DM, phase-transparent objects become visible by slightly defocusing an optical microscope operating in bright field mode [18, 19]. From the contrast generated by these objects (when they are out the focus), it is possible to obtain quantitative information about morphological, optical and mechanical properties of the cell [20–23]. The DM experiments were performed with a Nikon Eclipse TI inverted microscope equipped with a 610-nm band pass red filter, a stage-heated oil-immersion objective (Nikon Plan APO DIC H 100×, NA 1.3) (Nikon, Tokyo, NA 1.3, Nikon, Tokyo, Japan) and an environmental chamber (model Chamlide ICCU:109, Live Instrument, Nowan-gu, Korea), which provides a 37 °C environment. Images were captured using 8-bit CMOS (model 642M, Epix Inc, Buffalo Grove, IL, USA, 255 grey levels and 0.098 µm of pixel square side), with a gain of 8 db and a capture rate of 326 frames per second. The focal distance was controlled during the entire experiment by a Nikon Perfect Focus System apparatus. For each experiment, a volume of 0.05 µL of fresh blood (from the same healthy non-exposed volunteer used in the experiments described above) was resuspended in 1.0 mL of PBS containing 1 % (w/v) BSA and 500 µL of this mixture were transferred to a coverslip chamber. Each sample was prepared immediately prior to the experiment and was incubated at 37 °C for 15 min on the microscope stage in order to allow RBC deposition. Thereafter, single cell images of ten RBCs were continuously taken for 10 s. Purified antibodies, previously described here, were added at a final concentration of 2 % v/v followed by an incubation period of 30 min at 37 °C. An anti-bacteriophage monoclonal antibody produced in rabbit (Sigma-Aldrich), which binds specifically to phage coat proteins of fd phage or M13 phage, was used as control (non-related antibody). Subsequently, the same cells that had been analysed before were recorded for 10 s. All data were analysed using ImageJ *Plugins*, which corrects the background [23] and calculates the mean square contrast fluctuations of the defocused image in the asymptotic limit in a central area of the selected RBC of 1 µm × 1 µm approximately flat membrane, before and 30 min after the addition of each antibody [22].

### Statistical analysis

Data were analysed using GraphPad Prism 5.0 statistical software. D’Agostino-Pearson normality test was performed to evaluate if data sets fitted a Gauss distribution. For variables that displayed a normal distribution, the paired t test was used to compare the means of two matched groups, whereas one-way ANOVA followed by Tukey’s multiple comparison test was used to detect differences among the means of three or more unequal sample size groups. Data sets that did not display a normal distribution were analysed using non-parametric tests. The two-tailed Mann–Whitney U-test was used to compare the distributions of two unmatched groups, whereas the Kruskal–Wallis test followed by Dunn post hoc tests was used to compare three or more unpaired groups. Post hoc tests were run to provide specific information on which means were significantly different from each other. p values less than 0.05 were considered significant.

## Results

### IgG response to RBC antigens is different in patients with varied clinical status

**Fig. 1:**
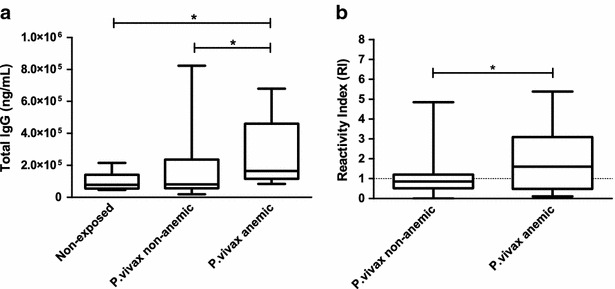
Associations between antibody responses and clinical status. **a** The concentrations of total IgG and **b** the levels of IgG against surface molecules of non-infected red blood cells (nRBCs) were evaluated in sera from healthy individuals (n = 11) and in sera from non-anaemic (n = 119) or anaemic *P*. *vivax*-infected patients (n = 11) by sandwich and cell-ELISA, respectively. The results are shown as median values and interquartile ranges. p values were determined using nonparametric tests: **a** Kruskal–Wallis followed by a post hoc Dunn’s multiple comparison test or **b** Mann–Whitney test. *Asterisks* indicate statistically significant difference (p value <0.05). Reactivity index (RI) was calculated as the ratio between the mean OD generated by each duplicate and the mean OD plus three standard deviations of samples from 11 malaria-naïve blood donors never exposed to malaria

Considering that hypergammaglobulinaemia and polyclonal B cell activation are common features during infections with *Plasmodium* [24–27], initially the concentration of total IgG in the serum of vivax patients was evaluated in relation to serum of healthy individuals. Non-anaemic vivax-infected patients and non-infected control subjects exhibited similar median concentrations of total IgG (p = 0.5956). However, the median concentration of total IgG in anaemic patients was significantly higher than the median concentration detected in non-anaemic infected subjects and healthy donors (p = 0.0041 and p = 0.0033, respectively) (Fig. 1a).

Next, the levels of IgG antibodies that recognize surface antigens of nRBCs were assessed. Figure 1b shows that anaemic patients infected with *P. vivax* had higher levels of IgG against erythrocyte molecules (median 1.61; interquartile range 0.48–3.09) when compared to IgG levels from infected non-anemic patients (median 0.86; interquartile range 0.52–1.20) (p = 0.0488).

### RBCs coated with IgG from anaemic patients are more susceptible to in vitro phagocytosis

**Fig. 2:**
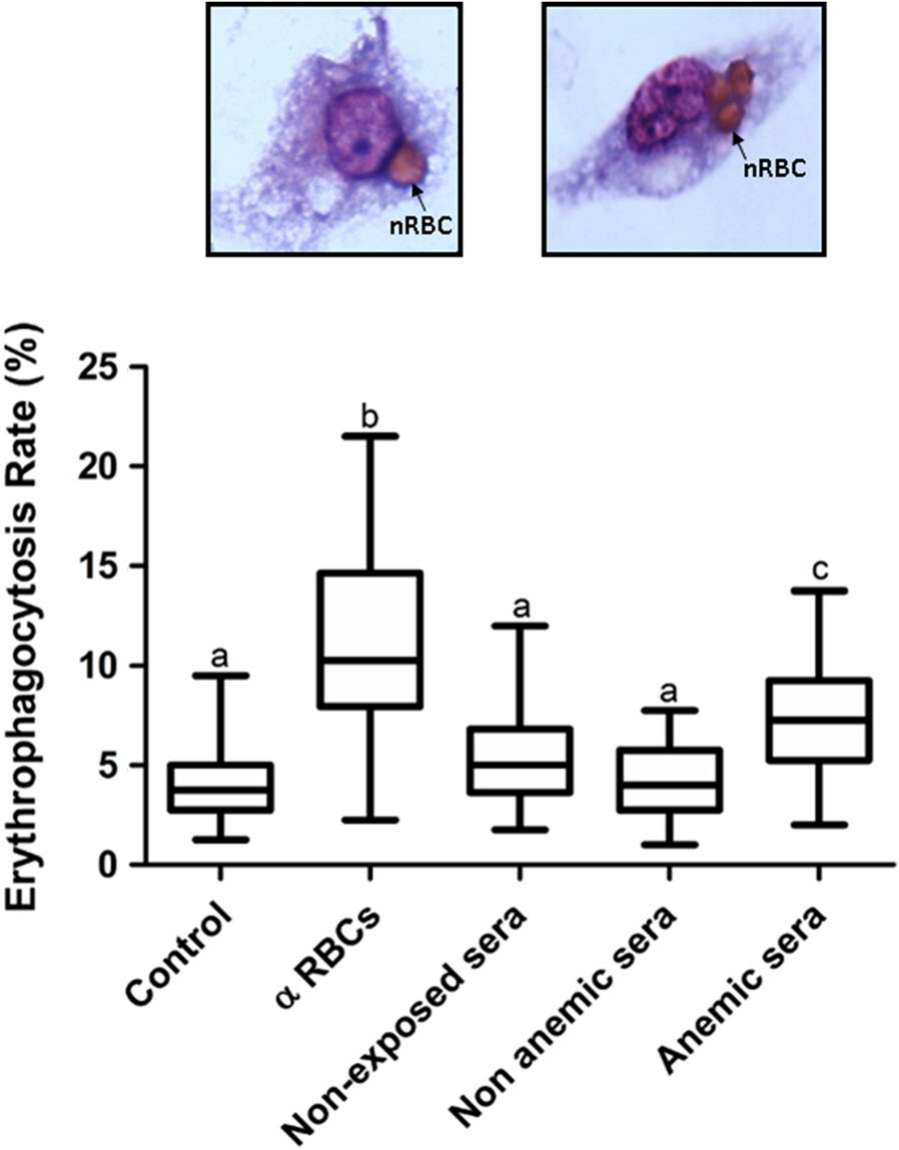
Evaluation of the phagocytic uptake, by THP-1 cells, of non-infected RBCs after their opsonization with different antibodies. nRBCs were isolated from a O^+^ healthy volunteer and after washing, they were opsonized with different antibodies: IgG against human red blood cells (α RBCs), IgG purified from healthy donors, IgG purified from non-anaemic patients with vivax malaria or IgG purified from patients with vivax malaria and anaemia. A group of nRBCs incubated with PBS was included for control. The combined result of six independent experiments is shown. Erythophagocytosis rate was calculated by counting the number of ingested nRBC on 400 phagocytic cells, under oil immersion light microscopy at 1000× magnification. Data are shown as box-and-whiskers plots, representing interquartile and complete ranges, with the horizontal line in each box indicating the median. p values were determined using ANOVA with Tukey’s post hoc test, in which all groups were pair-wised for statistical significance. *Alphabetic letters* above each box plot represent the results of Tukey’s multiple comparison test. Erythrophagocytosis rates that are not significantly different one from each other are represented with the same letter whereas unique (unshared) alphabetic letter indicate a significant different rate (p value <0.01). *Top* two representative light micrographs of ingested nRBCs within THP-1 activated monocytic cells

Because the removal of nRBCS by extravascular haemolysis is an important contributor to malarial anaemia [28–31], nRBCs were coated with immunoglobulins G purified from non-anaemic or anaemic vivax-infected patients to assess whether opsonization with these antibodies could induce RBC phagocytosis. Erythrophagocytosis rates reached their maximum levels in erythrocytes opsonized with polyclonal anti-human IgG directed to RBC molecules (up to three times higher than the basal levels observed in the control group). There were no significant differences among the mean values of phagocytic activity observed in assays with nRBCs opsonized with antibodies from non-anaemic patients (3.72 ± 2.30) compared to either the controls with non-opsonized RBCs (3.77 ± 2.51) or with RBCs coated with antibodies from healthy volunteers (3.40 ± 2.07). On the other hand, the phagocytic activity was higher for RBCs opsonized with IgG antibodies from anaemic patients (6.90 ± 3.23) in comparison to RBCs opsonized with IgG antibodies from the non-anemic subjects (Fig. 2).

### Opsonization of RBCs with anti-RBC antibodies decreases the dynamics of membrane fluctuations

**Fig. 3:**
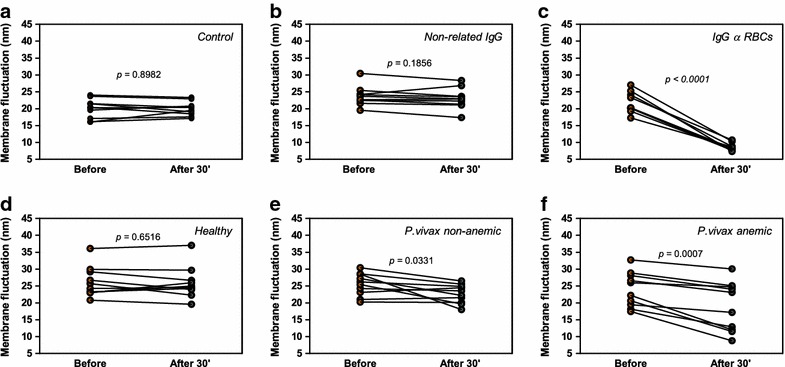
Effects of antibodies on the dynamics fluctuations of nRBC membrane. Modifications in the amplitude of membrane fluctuations of single human RBCs from a healthy donor were examined by DM before and 30 min after the addition of different antibodies: **a** control, **b** anti-bacteriophage monoclonal antibody produced in rabbit (non-related IgG), **c** anti-red blood cell antibody, **d** IgG purified from sera of healthy individuals, **e** IgG purified from sera of non-anaemic patients with vivax malaria, and **f** IgG purified from sera of subjects infected by *P*. *vivax* and with anaemia. For each assayed antibody, ten RBCs were evaluated. p values were determined using a paired t test

**Fig. 4:**
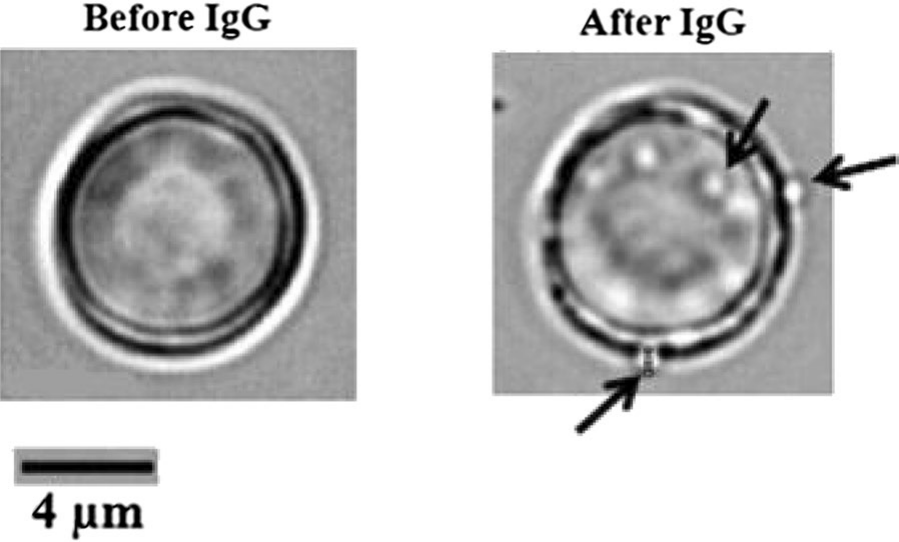
RBCs produce microparticles (indicated by *arrows*) after the addition of anti-red blood cell antibody (2 % v/v) in the cell suspension. The images were obtained using DM. A single human nRBC from a healthy donor was examined before and 30 min after the addition of the anti-red blood cell antibody

The increased levels of anti-nRBC antibodies found in the sera of anaemic patients and the ability of these sera to enhance phagocytosis led the authors to investigate the possibility of binding of such antibodies to nRBCs alters their dynamic membrane fluctuations. This is an important parameter to consider since changes in membrane fluctuations indicate alterations in the biomechanical properties of RBCs, such as the shear modulus, bending modulus and cytoplasmic viscosity [32–35]. Using DM, the membrane amplitude fluctuations of individual RBCs were determined before and after the addition of the different antibodies (Fig. 3). There was no significant difference (p = 0.8982) in the membrane fluctuation of each RBC from the control group before (20.04 ± 2.85 nm) and after 30 min (19.95 ± 2.06 nm) of observation, indicating that cell rigidity did not change during the period of experiment (Fig. 3a). An anti-bacteriophage monoclonal antibody (non-related IgG) was used to confirm that only antibodies specific to RBCs were able to modify the mechanical properties of these cells. The values of amplitude fluctuation in the RBC membranes before and after the addition of non-specific antibodies did not exhibit significant differences (23.67 ± 2.89 versus 23.04 ± 3.03 nm, respectively; p = 0.1856) (Fig. 3b). When RBCs were coated with the specific antibodies, a significant decrease in the amplitude of membrane fluctuation was observed when compared with the same but untreated RBCs. After addition of anti-RBC IgG, the mean membrane fluctuation of RBCs showed an overall reduction of 60 % (21.71 ± 3.06 to 8.56 ± 1.13 nm, p < 0.0001), suggesting a decrease in cell deformability (Fig. 3c). Moreover, a substantial amount of microvesicles (0.1–1.0 µm in size) shedding from nRBCs was observed after addition of this antibody (Fig. 4). When RBC membrane fluctuations were probed after the addition of antibodies from healthy individuals, no modification was observed (26.22 ± 4.50 nm before versus 25.90 ± 4.73 nm after the addition of IgG; p = 0.6516) (Fig. 3d). On the other hand, after the addition of antibodies purified from the sera of non-anaemic (Fig. 3e) and anaemic patients (Fig. 3f), reductions of approximately 11 % (25.50 ± 3.35 to 22.66 ± 2.80 nm; p = 0.0331) and 21 % (24.01 ± 5.16 to 18.95 ± 7.34 nm; p = 0.0007) were observed, respectively. Finally, it is important to mention that after the addition of each antibody no changes in RBC shape were observed.

## Discussion

Destruction of nRBCs is a pivotal aspect of malaria-induced anaemia but the immunological mechanisms involved in its genesis remain unknown, especially with regard to *P*. *vivax* infection. In order to shed some light in such mechanisms, the hypothesis that auto-antibodies directed to RBC antigens participate in the clearance of these cells was tested.

To investigate the potential role of antibodies in the destruction of nRBCs during vivax malaria, first the levels of total IgG and specific IgG against nRBC surface molecules were measured. Anaemic patients with *P.vivax* infection exhibited increased levels of both antibodies, suggesting that these immunoglobulins may play a harmful role in vivax malaria contributing to anaemia.

The presence of auto-antibodies against RBCs has been previously reported in vivax infections [13, 16]. Nevertheless, their role in the pathophysiology of anaemia has been little investigated. A recent study conducted with murine malaria demonstrated that anti-self phosphatidylserine antibodies generated during the plasmodial infection recognized nRBCs, contributing to anaemia in mice. In that study, it has also been shown that such antibodies are also detected in sera from patients with falciparum malaria, being increased levels of such immunoglobulins correlated with late post-malarial anaemia [36].

Auto-antibodies targeting RBCs can induce phagocytosis and complement-mediated cell lysis [37, 38] but their titres and specificity do not always manage to predict pathogenesis in infectious diseases. In these cases, assays involving phagocytes are useful to establish how well an antibody interacts with the clearance system. Accordingly, significantly higher in vitro phagocytic rates were observed for nRBCs opsonized with purified IgG from sera of anaemic patients in comparison to antibodies from the sera of non-anaemic patients and control healthy donors. These data suggest that the recognition of nRBC surface molecules by auto-antibodies, whose levels are increased during *P. vivax* infection, may play an important role in the removal of non-infected erythrocytes.

nRBC mechanical properties, which are associated with cell deformability and flexibility, were evaluated to further investigate the functional consequences of nRBC surface membrane antibody deposition. DM was used, for the first time, as a functional assay to measure the biomechanical modifications of RBCs after opsonization with purified IgG from patients with vivax infection. This technique has been shown to be a useful tool to evaluate membrane fluctuations in other biological systems, such as fibroblasts and macrophages [20, 39]. Interestingly, auto-antibodies from anaemic or non-anaemic vivax-infected patients decreased the nRBC membrane deformability, although this effect seems to be enhanced by auto-antibodies from anaemic vivax patients. In addition, it was demonstrated, using an immunoproteomic approach, that some protein spots of nRBC extract exclusively recognized by IgGs from anaemic patients with vivax malaria were identified by mass spectrometry as band 3 protein, an anion exchange mediator that is responsible for erythrocyte flexibility and shape maintenance (author, personal communication). From these results it can be speculated that the binding of such antibodies may modify the molecular conformation of erythrocyte membrane proteins, disturbing the interactions between the cytoskeleton and the lipid bilayer, leading to a reduction in RBC deformability and also leading to the removal of those cells by phagocytosis as it has been demonstrated by the binding of other extracellular ligands [40, 41].

The efficiency in RBC engulfment by macrophages is affected by physical properties (shape and stiffness) and molecular interactions at the cell surface [42, 43]. In the current study, no modifications in RBC shape were observed after the addition of different antibodies. On the other hand, an increase in RBC rigidity was reported after the opsonization with patients’ antibodies. This finding suggests that cell stiffness is the major contributor to nRBC clearance. It is possible that the rigidity of nRBCs opsonized with specific antibodies hyperactivates myosin-II of macrophages, stimulating the adhesion and enhancing the contractions of actin filaments, increasing RBC engulfment as previously reported [42].

Although the physical destruction of nRBCs mediated by auto-antibodies seems to be an important component of vivax-associated anaemia, a whole range of other possibilities [11, 44] cannot be excluded. Among them, the ineffective erythropoiesis/dyserithropoiesis, as judged by a variety of morphological abnormalities observed in erythroid precursor cells from bone-marrow aspirates obtained from *P. vivax*-infected patients [45]; the oxidative stress induced by metabolites generated during the infection, which may deplete RBC defense mechanisms, reducing their ability to protect themselves from the reactive oxygen species and rendering them more vulnerable to damage [46, 47]; the failure of nRBCs in recovering passage through splenic filter simulation, which leads to their destruction without phagocytosis [48], in addition to other factors.

It is worth mentioning that another interesting result obtained in the current study was the observation of microvesicles (0.1–1.0 µm in size) shedding from nRBCs after the addition of anti-RBC IgG but not after the incubation with purified antibodies from anemic patients. A possible explanation for this result is that anti-human RBC antibodies may induce an increased exposure of phosphatidylserine in the outer leaflet of red-blood cell membrane. Alternatively, antibodies from anaemic patients may bind to exposed phosphatidylserine and block the microvesicle formation. In either one condition or the other, free phosphatidylserine in outer membrane leaflet could be higher in the presence of polyclonal anti-human RBC IgG than in the presence of purified antibodies from anaemic patients. These speculations are not conclusive but they can be useful for further experimental investigations. Since increased levels of RBC microvesicles have been described in acute malaria as potential activators of the inflammatory responses [49–51] further investigation of the associations between antibodies and erythrocyte-derived microvesicles will allow a better understanding of their role in vivax anaemia pathogenesis.

## Conclusions

Anaemic patients with vivax malaria have increased levels of antibodies to RBCs and the deposition of these molecules on the erythrocyte surface decreases their deformability, predisposing them to clearance by phagocytic cells. These findings offer potential avenues to a better understanding of the immunopathological mechanisms involved in the destruction of nRBCs, leading to anaemia in *P. vivax* infections. Moreover, they will be important in the development of new therapy and vaccine strategies.

## Abbreviations

RBCs: red blood cells
nRBCs: non-infected red blood cells
DM: defocusing microscopy
PBS: phosphate buffered saline
HRP: horseradish peroxidase conjugated
OD: optical density
BSA: bovine serum albumin
OPD: *o*-phenylenediamine (benzene-1,2-diamine)
RI: reactivity index
THP-1: human Thp-1 monocytic leukaemia cells
